# Crossroads in virology: current challenges and future perspectives in the age of emerging viruses

**DOI:** 10.1242/dmm.050476

**Published:** 2023-09-20

**Authors:** Sumana Sanyal

**Affiliations:** Sir William Dunn School of Pathology, University of Oxford, South Parks Road, Oxford OX1 3RE, UK

**Keywords:** Host, Pathogen, Virology

## Abstract

Ongoing global health challenges posed by emerging and re-emerging viruses have highlighted the critical importance of understanding virus–host interactions in countering these threats. Environmental changes, urbanisation and ecological disruption, coupled with the adaptable nature of viruses, facilitates the emergence and spread of new viruses. This Editorial emphasises the urgency of a concerted effort in understanding virus–host interactions to inform the development of therapeutics and vaccines, and help predict disease outcomes. Furthermore, efforts to monitor viral evolution, identify mutations of concern, and develop ‘universal’ vaccines and broad-spectrum antiviral drugs are needed to counter viral evolution and potentially prevent future viral emergences. Widespread public mistrust surrounding viruses and vaccines also calls for improvement in science communication. A ‘One Health’ approach that advocates the development of robust global health systems, interdisciplinary collaborations and equity in health access is therefore imperative for transforming the virology landscape.

## Teaser

**Summary:** Understanding virus–host interactions at the microscopic and macroscopic scales is key to therapeutic and vaccine development, prediction of viral emergence and prevention of future outbreaks.

## How do new viruses emerge and persist?

Emerging and re-emerging viruses pose a significant global health challenge. They often appear without notice either due to introduction of vectors, such as mosquitoes, into a naïve human population, or due to spillovers from animals to humans, and can spread rapidly owing to the interconnected nature of the world. Examples of such pathogens include coronaviruses [e.g. severe acute respiratory syndrome coronavirus 2 (SARS-CoV-2)], flaviviruses (e.g. dengue, Zika), alphaviruses (e.g. chikungunya) and orthomyxoviruses (e.g. influenza). Emergence of new viruses is typically driven by several factors, including changes in land use, urbanisation and ecological disruption, all of which can alter the delicate balance between humans and wildlife, thereby increasing the likelihood of zoonotic transmissions. Repeated emergence of novel coronaviruses in the past two decades resulting in widespread outbreaks in human populations has been linked to spillovers from bats to humans via intermediary animals (Ruiz-Aravena et al., 2022). Climate change can also alter the geographic range of virus-carrying vectors, such as mosquitoes, leading to the emergence of viruses in new regions. The escalation of arboviruses – transmitted by mosquitoes and other arthropod vectors – in the Americas is causing high mortality among children and highlights the gravity of this public health emergency (Estallo et al., 2023).

Evolution of viral variants is often a key driver of persistence, with the coronavirus disease (COVID-19) pandemic serving as a potent reminder of the adaptability of viruses and associated challenges (Carabelli et al., 2023). Owing to the error-prone nature of viral polymerases, particularly among RNA viruses, mutations in their genomes can occasionally confer a selective advantage to the virus, such as increased transmissibility, virulence, immune evasion or the ability to infect a new host species. The emergence of SARS-CoV-2 variants from Alpha through to Omicron exemplifies the potential for viral evolution to dramatically alter disease dynamics. The success of each subsequent SARS-CoV-2 variant was enabled by altered intrinsic functional properties that increased transmissibility and, in some cases, caused partial resistance to neutralising antibodies, raising concerns about vaccine efficacy and re-igniting outbreaks. Monitoring viral evolution and immune signatures from infected patient samples in real time is therefore crucial (Budhraja et al., 2022), a task made feasible by advances in genomic and transcriptomic sequencing technologies. Equally important are laboratory-based studies that can help determine the functional impact of these mutations on virus behaviour and the effectiveness of existing countermeasures. To generate such knowledge, modelling infection in various genetically tractable *in vitro* and *in vivo* systems, such as in cell culture, organoids (van Der Vaart et al., 2021) and various animal models (Jeong et al., 2022; Leist et al., 2020), is paramount.

## Virus–host interactions inform therapy and prevention strategies

**Fig. 1.**
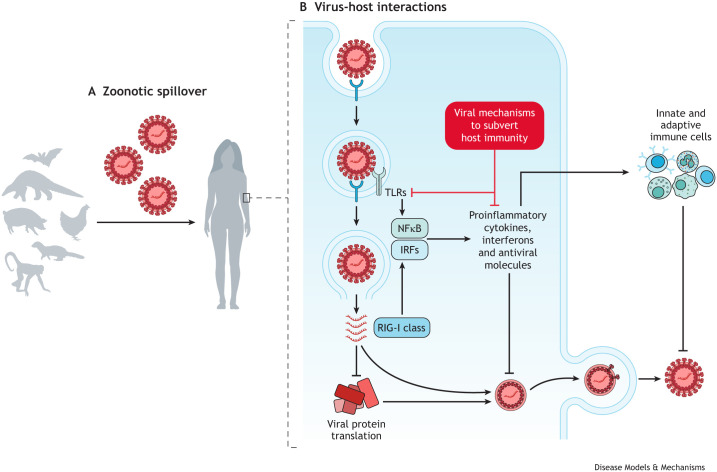
**Zoonotic spillover and virus–host interactions.** (A) Emerging and re-emerging viruses pose a significant global health challenge, in part due to zoonotic spillovers from animals to humans. (B) To combat the threat of these ever-evolving viral pathogens, researchers must understand the interconnected viral mechanisms and host defence responses. The intracellular replication of an RNA virus is shown here in red. Host TLRs and RIG-I class receptors detect the intracellular virus and activate transcription factors NFκB and IRFs, which initiate the production of proinflammatory cytokines, interferons and antiviral molecules. This directly inhibits viral replication and recruits innate and adaptive immune cells to help clear the viral infection. Consequently, viruses have developed mechanisms to subvert these host immune responses. IRF, interferon response factor; NFκB, nuclear factor-κB; RIG-I, retinoic acid-inducible gene I; TLR, Toll-like receptor.

A key facet that holds enormous promise in our collective battle against viral diseases is a molecular understanding of virus–host interactions. These interactions can illuminate the complex interplay of biological mechanisms that determine the course of a viral infection (Fig. 1). At the most fundamental level, a virus needs to recognise and bind to specific host cell receptors, infiltrate the cell, and exploit host molecular machinery to replicate, assemble into progenies and exit to spread into neighbouring cells. Each of these steps is a potential target for therapeutic intervention, and studies in various infection models have yielded important insights into viral pathogenesis and therapeutic targets. For instance, the discovery of distinct viral entry routes of the alphaviruses chikungunya and Sindbis was made possible using zebrafish as a model system, and has shaped our understanding of neuroinvasion and resulting encephalopathies (Passoni et al., 2017). Similarly, neurological abnormalities triggered by Zika virus was successfully recapitulated in *Drosophila* (Harsh et al., 2020), paving the way for targeted interventions.

In the same vein, host responses to viral infections, particularly those of the immune system, are critical determinants of disease outcomes. Following virus exposure, a complex interplay of B cells, T cells and other immune factors supports immunological memory, a cornerstone of vaccine-induced protection. For example, neuroinflammation triggered by Japanese encephalitis virus, a mosquito-borne flavivirus with expanding incidence and geographical range, was recently reported in a mouse model (Tripathi et al., 2021), facilitating molecular characterisation of the viral-induced immunopathology. Understanding host immune responses to viral infection, both innate and adaptive, can guide the development of vaccines and immunotherapies.

Viruses, in turn, have co-evolved diverse strategies to evade or subvert host immunity. These interactions therefore form the frontline in the ongoing evolutionary arms race between viruses and their hosts. Some viruses can downregulate host cell surface proteins to effectively hide from immune surveillance. Others can interfere with signalling pathways involved in the production of interferon-stimulated genes or with post-translational modifications of host proteins that affect the function of antiviral molecules (Munnur et al., 2021, 2022). Host enzymes regulating such modifications are routinely targeted by viruses and therefore remain an exciting area for drug discovery (Marín-Rubio et al., 2023). The role of the microbiome in modulating virus–host interactions is also an emerging area of research. Changes in the microbiome have been shown to alter host immunity in *Drosophila* and mouse models (Wong et al., 2016), thereby influencing susceptibility to viral replication, with effects on disease severity and transmission.

Understanding virus–host interactions is equally critical for predicting and preventing zoonotic spillovers. The ability of a virus to infect a new host species depends on compatible virus–host interactions at multiple levels. Insights into these interactions can inform risk assessments of potential zoonotic viruses and guide surveillance efforts. Therefore, deconstructing mechanisms and functional implications of these interactions in simpler model systems with evolutionarily conserved pathways is essential to effectively tackle emerging infections (Bier and Guichard, 2012). Such knowledge can also guide the development of therapeutics and vaccines, inform public health strategies, and potentially even predict and prevent future virus emergences (Henderson Sousa et al., 2023). As virologists, immunologists, geneticists and other researchers continue to unravel the complex interplay between viruses and their hosts, we can expect exciting breakthroughs in our ongoing struggle against these pathogens.

## Combatting viral emergence and evolution

A key challenge in dealing with emerging and re-emerging viruses is their unpredictability. Our knowledge of these viruses is often limited at the outset, hampering timely and effective responses. Hence, proactive surveillance in both human and animal populations, together with a robust global public health infrastructure, are critical. Furthermore, research into viral genomics, evolution, ecology and host interactions of these viruses is crucial. Understanding these aspects can shed light on virus origins and predict potential transmission dynamics, providing valuable information for the prevention, control and treatment of these diseases. The recurring pattern of viral emergence has highlighted the need for a sustained, integrated ‘One Health’ approach, recognising the interconnectedness of human, animal and environmental health. It is imperative for the global scientific community to tackle this challenge, ensuring our preparedness and resilience in the face of future viral threats.

Vaccines are, of course, an instrumental strategy in controlling infectious diseases. However, their efficacy can often be compromised on account of viral evolution, such as with influenza virus, which necessitates annual vaccine updates. In other cases, such as dengue, developing an effective vaccine has proved challenging on account of the complex interactions of the pathogen with the immune system (van Leur et al., 2021). Although these examples underscore the need for ‘universal’ vaccines that protect against a wide range of strains within a viral species, development of broad-spectrum antiviral drugs is another important strategy in countering viral evolution. These drugs would ideally target conserved viral components or host factors necessary for viral replication, making them effective against multiple viral species and less prone to generating resistance.

## Barriers beyond biology

The rapid development of the COVID-19 vaccine and current pursuit of universal vaccines has raised the concern of global health inequity. Differences in vaccination rates due to logistical issues, resource allocation and economic disparities between low- and high-income countries exacerbate the problem, often prolonging the path to herd immunity. The ability to scale up vaccination campaigns is also challenging in countries with less developed healthcare infrastructure.

Another daunting challenge we currently face that lies not solely within the biological realm is the lack of public understanding and trust in virology. In this era of rapid information exchange, misinformation about viruses and vaccines spreads almost as quickly as the pathogens, exacerbating the difficulty of managing public health crises. There is a growing disconnect between scientific findings and public understanding, often fuelled by the rapid circulation of incorrect or misleading information. Particularly in the context of the COVID-19 pandemic, misconceptions about virus transmission, disease severity and vaccine safety have proliferated across social media and other communication platforms. These circumstances have resulted in public mistrust in science and hesitancy towards vaccinations, posing significant barriers to disease control efforts (van Der Linden, 2022). It is the responsibility of open-access journals, like Disease Models & Mechanisms, to ensure that published science investigating virology is robust and thoroughly reviewed by experts. And as researchers, we should strive to clearly communicate our science to the scientific community and to the public.

Virology stands at the crossroads of promise and peril. The challenges posed by emerging viruses, evolving variants, global vaccine inequity and public misinformation present significant concerns. Nevertheless, advances in viral surveillance, genomics, immunology and therapeutics provide sufficient reasons for optimism. We must aim for novel solutions not only to treat and prevent viral infections, but also to predict and forestall future emergences as we continue to unravel the intricate dynamics between viruses, hosts and their environment.
